# Supplementary material for: Steller’s sea cow genome suggests this species began going extinct before the arrival of Paleolithic humans
Source: Nat Commun. 2021 Apr 13;12:2215. doi: 10.1038/s41467-021-22567-5 (PMC8044168; doi:10.1038/s41467-021-22567-5)
Supplement: Supplementary file 1 — Supplementary Information [file 41467_2021_22567_MOESM1_ESM.docx]

**Supplementary Information for**

**Steller's sea cow genome suggests this species began going extinct before the arrival of Paleolithic humans**

Fedor S. Sharko^1,2,†^, Eugenia S. Boul­­­ygina^1,†^, Svetlana V. Tsygankova^1^, Natalia V. Slobodova^1^, Dmitry A. Alekseev ^3^, Anna A. Krasivskaya^4^, Sergey M. Rastorguev^1^, Alexei N. Tikhonov^5,6^, Artem V. Nedoluzhko^7,^*

^1^ National Research Center "Kurchatov Institute", 1st Akademika Kurchatova Square, 123182 Moscow, Russia.

^2^ Research Center of Biotechnology of the Russian Academy of Sciences, Leninsky prospect 33/2, 119071 Moscow, Russia.

^3^ Russian Presidential Academy of National Economy and Public Administration, Prospect Vernadskogo, 82, 119571, Moscow, Russia.

^4^ Skolkovo Institute of Science and Technology, 143028, Moscow, Russia.

^5^ Zoological Institute Russian Academy of Sciences, Universitetskaya nab., 1, 199034, Saint-Petersburg, Russia.

^6^ Institute of Applied Ecology of the North, North-Eastern Federal University, Lenina 43, 677980, Yakutsk, Russia.

^7^ Faculty of Biosciences and Aquaculture, Nord University, 8049, Bodø, Norway.

* Correspondence: artem.nedoluzhko@nord.no; Tel.: +47 469–01–369

^†^ Equally contributed

Supplementary Table 1. Illumina generated reads and number of mapped reads to West Indian Manatee (*Trichechus manatus*) reference genome sequence (GCA_000243295.1) for the test Illumina sequencing run

| Library name | Total number of reads | Number of contaminant  reads | Number of reads after PALEOMIX quality filtration | Total number of mapped reads | The ratio of mapped reads | Reads clonality | Coverage, fold | Number of endogenus reads, % | Breadth of coverage | Average read length |
| --- | --- | --- | --- | --- | --- | --- | --- | --- | --- | --- |
| Lib1k | 6,531,468 | 239,109 | 3,541,664 | 1,622,287 | 0.458 | 0.0428 | 0.067 | 45.80% | 6,43% | 135.75 |
| Lib3k | 6,608,042 | 178,933 | 3,672,407 | 1,730,284 | 0.4711 | 0.035 | 0.074 | 47.11% | 7,00% | 139 |
| Lib4-2 | 7,544,222 | 158,570 | 4,152,543 | 1,671,316 | 0.4024 | 0.0359 | 0.074 | 40.32% | 6,95% | 142.95 |
| Lib4-1 | 7,810,724 | 173,112 | 4,268,068 | 1,721,048 | 0.4032 | 0.034 | 0.074 | 40.24% | 6,91% | 138.35 |
| St11 | 7,117,656 | 147,437 | 3,709,542 | 1,550,338 | 0.4179 | 0.0484 | 0.066 | 41.79% | 6,31% | 140.83 |
| St12 | 5,993,680 | 172,576 | 3,266,363 | 851,541 | 0.26 | 0.039 | 0.039 | 26.07% | 3,76% | 149.89 |

Supplementary Table 2. Illumina generated reads and number of mapped reads to West Indian Manatee (*Trichechus manatus*) reference genome sequence (GCA_000243295.1) for the deep Illumina sequencing run

| Library name | Total number of reads | Number of contaminant  reads | Number of reads after PALEOMIX quality filtration | Total number of mapped reads | The ratio of mapped reads | Reads clonality | Coverage, fold | Average read length | Number of endogenous reads, % |
| --- | --- | --- | --- | --- | --- | --- | --- | --- | --- |
| Lib1k | 412,855,578 | 7,213,755 | 370,704,842 | 197,716,234 | 0.5333 | 0.4682 | 1.86 | 55.15 | 53.33% |
| Lib3k | 430,047,500 | 6,200,196 | 390,655,189 | 224,632,611 | 0.575 | 0.2256 | 3.06 | 54.77 | 57.50% |
| Lib4-2 | 480,168,784 | 4,743,095 | 445,753,846 | 246,842,499 | 0.5537 | 0.1784 | 3.5 | 53.7 | 55.37% |
| St11 | 434,161,498 | 4,088,598 | 398,019,593 | 214,909,446 | 0.5399 | 0.3606 | 2.4 | 54.26 | 53.99% |

Supplementary Table 3. Gene ontology analysis for loci with nonsense and non-synonymous variants. *p-value* < 0.01

| Nonsense variants | | |
| --- | --- | --- |
| GO term | **Description** | ***p-value*** |
| Biological processes | | |
| GO:0006633 | fatty acid biosynthetic process | 2.5E-4 |
| GO:0023052 | signaling | 4.84E-4 |
| Non-synonymous variants | | |
| Biological processes | | |
| GO:0050877 | nervous system process | 1.06E-4 |
| GO:1903510 | mucopolysaccharide metabolic process | 1.15E-4 |
| GO:0030203 | glycosaminoglycan metabolic process | 1.22E-4 |
| GO:0007600 | sensory perception | 1.4E-4 |
| GO:0099550 | trans-synaptic signaling, modulating synaptic transmission | 1.92E-4 |
| GO:0006022 | aminoglycan metabolic process | 2.31E-4 |
| GO:0097400 | interleukin-17-mediated signaling pathway | 3.62E-4 |
| GO:0018094 | protein polyglycylation | 3.87E-4 |
| GO:0006027 | glycosaminoglycan catabolic process | 5.11E-4 |
| GO:0030212 | hyaluronan metabolic process | 6.2E-4 |
| GO:0016998 | cell wall macromolecule catabolic process | 6.75E-4 |
| GO:0044036 | cell wall macromolecule metabolic process | 6.75E-4 |
| Molecular function | | |
| GO:0030368 | interleukin-17 receptor activity | 3.62E-4 |
| GO:0070735 | protein-glycine ligase activity | 3.87E-4 |
| GO:0070736 | protein-glycine ligase activity, initiating | 3.87E-4 |
| GO:0008536 | Ran GTPase binding | 6.14E-4 |
| GO:0003796 | lysozyme activity | 6.75E-4 |

Supplementary Table 4. Genome-wide heterozygosity in the extinct Late Pleistocene (Oimyakon) and Middle Holocene (Wrangel) woolly mammoth populations, Steller’s sea cow population as well as modern animal populations: dugong, narwhal, polar bear, walrus, and beluga whale. (θ): population mutation rate which approximates heterozygosity under the infinite sites model, (ε) : sequencing error estimated by mlRho v.2.9, CI: confidence interval.

|  | θ | CI (θ) | ε | CI (ε) |
| --- | --- | --- | --- | --- |
| Wrangel mammoth | 1.03 | 1.00-1.05 | 0.897 | 0.896-0.888 |
| Oimyakon mammoth | 1.25 | 1.21-1.26 | 0.913 | 0.910-0.914 |
| Steller’s sea cow | 1.19 | 1.18-1.19 | 1.63 | 1.63-1.63 |
| Dugong | 2.19 | 2.17-2.21 | 1.36 | 1.36-1.37 |
| Narwhal | 0.49 | 0.49-0.50 | 0.403 | 0.402-0.404 |
| Polar bear | 0.435 | 0.430-0.440 | 0.652 | 0.651-0.654 |
| Walrus | 0.634 | 0.624-0.645 | 0.807 | 0.802-0.811 |
| Beluga whale | 0.962 | 0.956-0.968 | 0.889 | 0.888-0.891 |

Supplementary Table 5. Gene ontology analysis of the loci/genes which were under positive selection in *H. gigas* genome (dN/dS threshold > 1). *p-value* < 0.01

| GO term | Description | *p-value* |
| --- | --- | --- |
| Biological processes | | |
| GO:0090278 | negative regulation of peptide hormone secretion | 2.19E-4 |
| GO:0099010 | modification of postsynaptic structure | 2.95E-4 |
| GO:0098885 | modification of postsynaptic actin cytoskeleton | 2.95E-4 |
| GO:0060074 | synapse maturation | 2.95E-4 |
| GO:0006952 | defense response | 5.07E-4 |
| GO:0035809 | regulation of urine volume | 5.74E-4 |
| GO:0035810 | positive regulation of urine volume | 5.74E-4 |
| GO:0090501 | RNA phosphodiester bond hydrolysis | 6.42E-4 |
| GO:0046325 | negative regulation of glucose import | 6.76E-4 |
| Molecular function | | |
| GO:0048018 | receptor ligand activity | 3.5E-4 |
| GO:0005102 | signaling receptor binding | 3.8E-4 |
| GO:0030545 | receptor regulator activity | 4.51E-4 |
| GO:0001784 | phosphotyrosine residue binding | 4.53E-4 |
| GO:0045309 | protein phosphorylated amino acid binding | 4.53E-4 |

Supplementary Table 6. Gene ontology analysis of the loci/genes which were under positive selection in *H. gigas* genome (dN/dS threshold > 3), *p-value* < 0.01

| GO term | Description | *p-value* |
| --- | --- | --- |
| Biological processes | | |
| GO:0006952 | defense response | 1.8E-05 |
| Molecular function | | |
| GO:0048018 | receptor ligand activity | 5.5E-04 |
| GO:0005102 | signaling receptor binding | 4.6E-03 |
| GO:0030545 | receptor regulator activity | 1.6E-03 |

Supplementary Table 7. Mapping statistics for *H. gigas* reads aligned to African elephant reference genome (Loxafr4). Chromosome 8 and chromosome X marked by grey

| Chromosome number | Total number of mapped reads | Depth of coverage, % |
| --- | --- | --- |
| chr1 | 27,218,576 | 51.89 |
| chr2 | 28,678,722 | 52.21 |
| chr3 | 26,701,102 | 53.28 |
| chr4 | 22,195,143 | 53.25 |
| chr5 | 17,375,699 | 51.15 |
| chr6 | 18,516,703 | 49.64 |
| chr7 | 10,024,800 | 53.58 |
| chr8 | 15,702,033 | 51.67 |
| chr9 | 11,014,759 | 51.03 |
| chr10 | 12,849,036 | 53.15 |
| chr11 | 8,471,089 | 51.02 |
| chr12 | 9,429,423 | 46.68 |
| chr13 | 13,393,800 | 50.88 |
| chr14 | 8,974,230 | 47.89 |
| chr15 | 10,178,545 | 49.99 |
| chr16 | 5,600,312 | 45.40 |
| chr17 | 8,176,044 | 49.51 |
| chr18 | 9,200,691 | 51.69 |
| chr19 | 8,089,924 | 49.78 |
| chr20 | 8,711,657 | 46.97 |
| chr21 | 6,899,845 | 47.54 |
| chr22 | 5,840,281 | 47.90 |
| chr23 | 7,180,213 | 50.42 |
| chr24 | 2,488,287 | 41.83 |
| chr25 | 7,471,987 | 48.77 |
| chr26 | 8,666,599 | 46.51 |
| chr27 | 6,720,519 | 47.59 |
| chrX | 19,908,994 | 53.22 |

Supplementary Table 8. Holocene archeological sites in Bering sea region where bone samples of Steller’s sea cow were described

| # | Archeological site location | Dates | Reference |
| --- | --- | --- | --- |
| 1 | Aleutian Islands, Adak Island (ADK-009 site) | 1710±70 BCE | ^1^ |
| 2 | Aleutian Islands, Buldir Island | 1611±67 BCE | ^1^ |
| 3 | Alaska, Noatak river | XVI century | ^2^ |
| 4 | Aleutian Islands, Attu Island | ? | ^3^ |
| 5 | Aleutian Islands, Kiska Island | 1000 BCE or XVII-XVIII century | ^3^ |
| 6 | St. Lawrence Island | 800±900 BCE | ^4^ |

Supplementary Table 9. Reference genomes and sequencing data of extinct and extant mammal species which were used for comparative demography structure analysis of Steller’s sea cow

| Species | Reference genome, NCBI accession | Reads, SRA accession |
| --- | --- | --- |
| Beluga whale – *Delphinapterus leucas* | GCA_002288925.2 | SRR5659909 |
| Polar bear – *Ursus maritimus* | GCF_000687225.1 | SRR942309 |
| Walrus – *Odobenus rosmarus* | GCF_000321225.1 | SRR575505 |
| Narwhal – *Monodon monoceros* | GCA_005125345.1 | SRR8284578 |
| Woolly mammoth – *Mammuthus primigenius* (Oimyakon) | Loxafr3.0 (GCA_000001905.1) | ERR852028 |
| Woolly mammoth – *Mammuthus primigenius* (Wrangel) | Loxafr3.0 (GCA_000001905.1) | ERR855944 |
| Pleistocene Lena horse - *Equus lenensis* | EquCab3.0 (GCA_002863925.1) | SRR1824455 |
| Dugong – *Dugong dugon* | GCA_000243295.1 | DRR251525 |

Supplementary Table 10. Mutation rates which were used for demography structure analysis of Steller’s sea cow. Mutation rates for *Delphinapterus leucas*, *Ursus maritimus, Odobenus rosmarus*, *Monodon monoceros* were obtained from Westbury et al. *^5^* Mutation rates for two woolly mammoth specimens were obtained from Palkopoulou et al. ^6^ Mutation rate for Pleistocene Lena horse was obtained from Schubert et al. ^7^

| Species | Generation time | Mutation rate |
| --- | --- | --- |
| Beluga whale – *Delphinapterus leucas* | 32 | 1.65e-08 |
| Polar bear – *Ursus maritimus* | 11.2 | 1.83e-08 |
| Walrus – *Odobenus rosmarus* | 15 | 9.40e-09 |
| Narwhal *– Monodon monoceros* | 30 | 1.56e-08 |
| Steller’s sea cow – *Hydrodamalis gigas* | 27 | 1.19e-09 |
| Woolly mammoth – *Mammuthus primigenius* (Oimyakon) | 31 | 1.25e-09 |
| Woolly mammoth – *Mammuthus primigenius* (Wrangel) | 31 | 1e-09 |
| Pleistocene Lena horse - *Equus lenensis* | 8 | 7.24e-09 |
| Dugong – *Dugong dugon* | 27 | 1.19e-09 |

**Supplementary Figures**

Supplementary Figure 1. Postmortem DNA damage patterns in the *H. gigas* DNA-library Lib1k. Damage patterns of DNA library generated by MapDamage 2.0 present C to T (and complementary G to A) misincorporations at the 5’ and 3’ termini of the last 25 nucleotides. The Y-axis shows frequency of misincorporations. The X-axis shows terminal 25 nucleotides of DNA library.

Supplementary Figure 2. Postmortem DNA damage patterns in the *H. gigas* DNA-library Lib3k. Damage patterns of DNA library generated by MapDamage 2.0 present C to T (and complementary G to A) misincorporations at the 5’ and 3’ termini of the last 25 nucleotides. The Y-axis shows frequency of misincorporations. The X-axis shows terminal 25 nucleotides of DNA library.

Supplementary Figure 3. Postmortem DNA damage patterns in the *H. gigas* DNA-library Lib4-2. Damage patterns of DNA library generated by MapDamage 2.0 present C to T (and complementary G to A) misincorporations at the 5’ and 3’ termini of the last 25 nucleotides. The Y-axis shows frequency of misincorporations. The X-axis shows terminal 25 nucleotides of DNA library.

Supplementary Figure 4. Postmortem DNA damage patterns in the *H. gigas* DNA-library St11. Damage patterns of DNA library generated by MapDamage 2.0 present C to T (and complementary G to A) misincorporations at the 5’ and 3’ termini of the last 25 nucleotides. The Y-axis shows frequency of misincorporations. The X-axis shows terminal 25 nucleotides of DNA library.

Supplementary Figure 5. Maximum likelihood phylogenetic tree reconstruction of the Tethytheria species, including the extinct Steller’s sea cow based on nuclear genome sequences, and rooted with a rock hyrax outgroup. Created with BioRender.com.


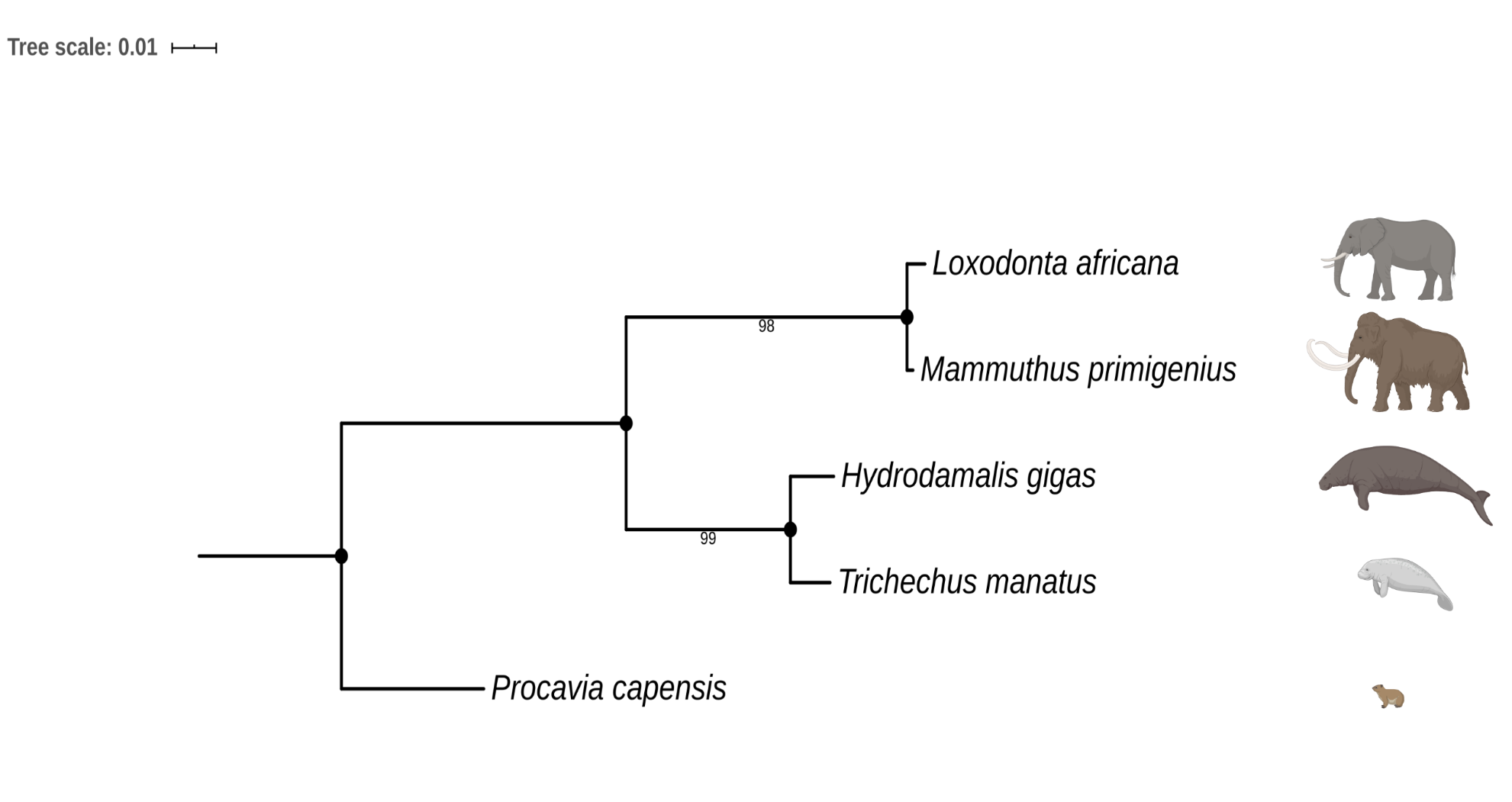


Supplementary Figure 6. Demographic history of Steller’s sea cow and modern marine mammals. Time is given in units of divergence per base pair on the X-axis, effective population size is shown on the Y-axis. Analyze the support for the resultant PSMC analysis with 100 bootstrap replicates for *H. gigas* (A), *D. dugon* (F), and modern Arctic marine mammals (B-E). Population size history of each species marked by red color.


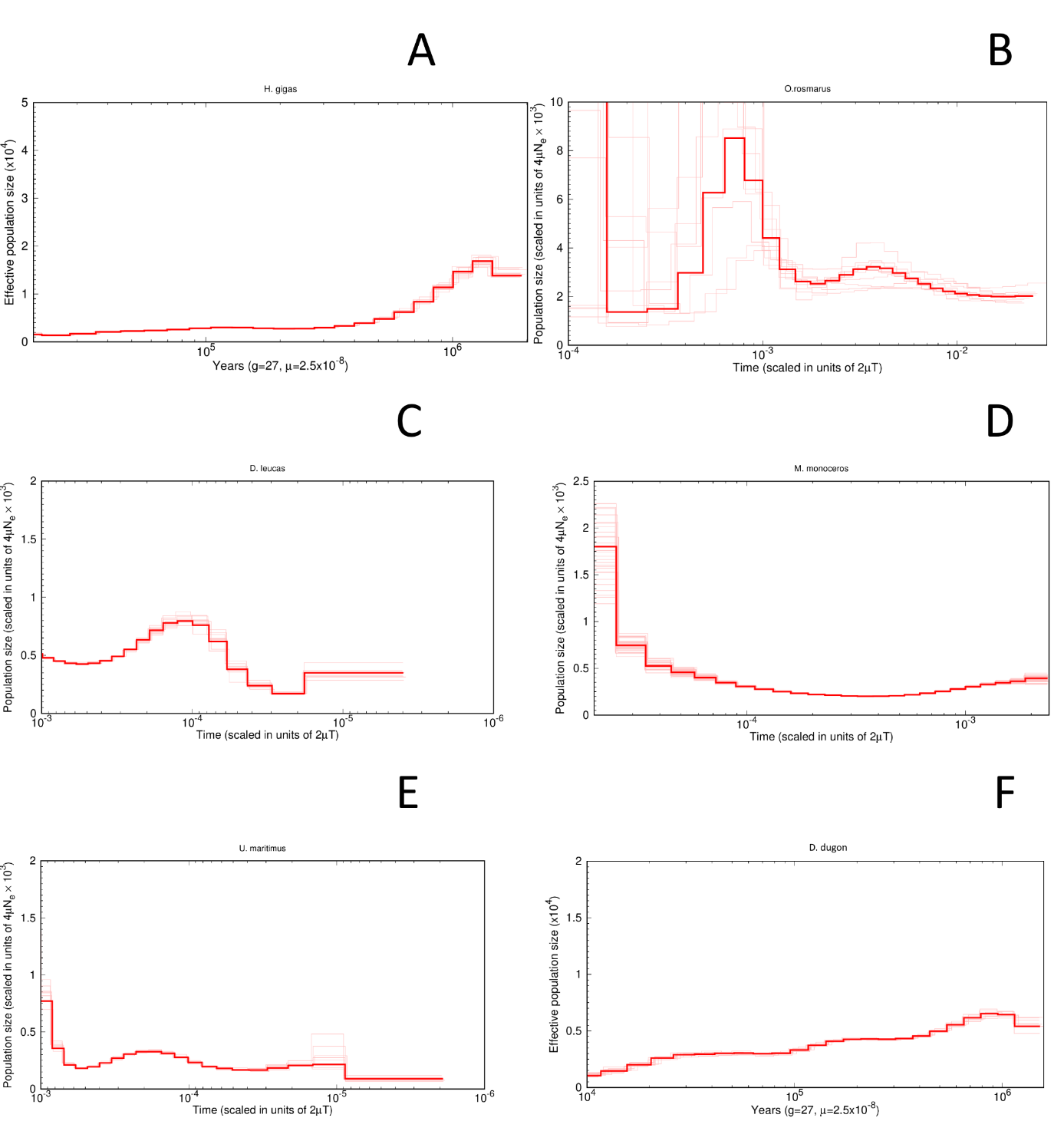


Supplementary Figure 7. Demographic history of Steller’s sea cow and modern marine mammals. Time is given in units of divergence per base pair on the X-axis, effective population size is shown on the Y-axis. Comparative Steller’s sea cow, dugong, and modern Arctic species effective population size history inferred using the PSMC model. Population size history of each species marked by different color.


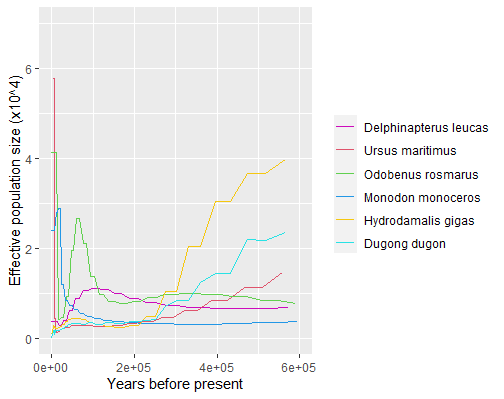


**References**

1 Savinetsky, A. B., Kiseleva, N. K. & Khassanov, B. F. Dynamics of sea mammal and bird populations of the Bering Sea region over the last several millennia. *Palaeogeography, Palaeoclimatology, Palaeoecology* **20**, 335-352 (2004).

2 Whitmore, F. C. & Gard, L. M. J. Steller’s sea cow (Hydrodamalis gigas) of late Pleistocene age from Amchitka, Aleutian Islands, Alaska. *U.S. Geological Survey Professional Paper* **1036**, 1-19 (1977).

3 Domning, D. P., Thomason, J. & Corbett, D. G. Steller's sea cow in the Aleutian Islands. *Marine Mammal Science* **23**, 976-983 (2007).

4 Crerar, L. D., Crerar, A. P., Domning, D. P. & Parsons, E. C. Rewriting the history of an extinction-was a population of Steller's sea cows (Hydrodamalis gigas) at St Lawrence Island also driven to extinction? *Biol Lett* **10**, 20140878, doi:10.1098/rsbl.2014.0878 (2014).

5 Westbury, M. V., Petersen, B., Garde, E., Heide-Jorgensen, M. P. & Lorenzen, E. D. Narwhal Genome Reveals Long-Term Low Genetic Diversity despite Current Large Abundance Size. *iScience* **15**, 592-599, doi:10.1016/j.isci.2019.03.023 (2019).

6 Palkopoulou, E. *et al.* Complete genomes reveal signatures of demographic and genetic declines in the woolly mammoth. *Curr Biol* **25**, 1395-1400, doi:10.1016/j.cub.2015.04.007 (2015).

7 Schubert, M. *et al.* Prehistoric genomes reveal the genetic foundation and cost of horse domestication. *Proc Natl Acad Sci U S A* **111**, E5661-5669, doi:10.1073/pnas.1416991111 (2014).
