# Supplementary material for: Steller’s sea cow genome suggests this species began going extinct before the arrival of Paleolithic humans
Source: Nat Commun. 2021 Apr 13;12:2215. doi: 10.1038/s41467-021-22567-5 (PMC8044168; doi:10.1038/s41467-021-22567-5)
Supplement: Supplementary file 3 — Reporting Summary [file 41467_2021_22567_MOESM3_ESM.pdf]

nature research

Corresponding author(s): Artem Nedoluzhko

Last updated by author(s): Mar 1, 2021

Reporting Summary

Nature Research wishes to improve the reproducibility of the work that we publish. This form provides structure for consistency and transparency in reporting. For further information on Nature Research policies, see our Editorial Policies and the Editorial Policy Checklist.

Statistics

For all statistical analyses, confirm that the following items are present in the figure legend, table legend, main text, or Methods section.

n/a	Confirmed	
☐	☒	The exact sample size (*n*) for each experimental group/condition, given as a discrete number and unit of measurement
☒	☐	A statement on whether measurements were taken from distinct samples or whether the same sample was measured repeatedly
☐	☒	The statistical test(s) used AND whether they are one- or two-sided<br>*Only common tests should be described solely by name; describe more complex techniques in the Methods section.*
☒	☐	A description of all covariates tested
☐	☒	A description of any assumptions or corrections, such as tests of normality and adjustment for multiple comparisons
☐	☒	A full description of the statistical parameters including central tendency (e.g. means) or other basic estimates (e.g. regression coefficient) AND variation (e.g. standard deviation) or associated estimates of uncertainty (e.g. confidence intervals)
☒	☐	For null hypothesis testing, the test statistic (e.g. *F*, *t*, *r*) with confidence intervals, effect sizes, degrees of freedom and *P* value noted<br>*Give P values as exact values whenever suitable.*
☒	☐	For Bayesian analysis, information on the choice of priors and Markov chain Monte Carlo settings
☒	☐	For hierarchical and complex designs, identification of the appropriate level for tests and full reporting of outcomes
☒	☐	Estimates of effect sizes (e.g. Cohen's *d*, Pearson's *r*), indicating how they were calculated

Our web collection on statistics for biologists contains articles on many of the points above.

Software and code

Policy information about availability of computer code

Data collection	n/a
Data analysis	Only previously published tools were used in this study: PALEOMIX v1.2.14; BWA v0.7.17; Mapdamage v2.0; BBmap v38.49; BBDuk v38.49; Spades v3.10; blastn v2.7.1+; Samtools v1.7; PSMC v0.6.5; RAxML v8.2.12; BUSCO v4.0.5; Muscle v3.8.31; mlRho v2.9; SnpEff v4.3; BCFtools v1.9; Gorilla v1; SNPGenie v2019.10.31; MITOS v2; GeSeq v1.84; TOL v4; MUSCLE v.3.8.31; ASTRAL v5.7.2; ANGSD v0.930

For manuscripts utilizing custom algorithms or software that are central to the research but not yet described in published literature, software must be made available to editors and reviewers. We strongly encourage code deposition in a community repository (e.g. GitHub). See the Nature Research guidelines for submitting code & software for further information.

Data

Policy information about availability of data

All manuscripts must include a data availability statement. This statement should provide the following information, where applicable:
- Accession codes, unique identifiers, or web links for publicly available datasets
- A list of figures that have associated raw data
- A description of any restrictions on data availability

(1) Databases:
Mammalia odb10 database: https://busco-data.ezlab.org/v4/data/
(2) Nuclear genomes:
Steller's sea cow (this study) genomic data: National Center for Biotechnology Information
Pleistocene Lena horse genomic data: https://www.ncbi.nlm.nih.gov/bioproject/PRJEB7537
Woolly mammoth genomic data: https://www.ncbi.nlm.nih.gov/bioproject/PRJEB7929/

African bush elephant genomic data (LoxAfr3.0): https://www.ncbi.nlm.nih.gov/assembly/GCF_000785645.1
African bush elephant genomic data (LoxAfr4): ftp://ftp.broadinstitute.org/pub/assemblies/mammals/elephant/loxAfr4/
Dugong genomic data: https://www.ncbi.nlm.nih.gov/sra/DRR251525
West Indian Manatee genomic data: https://www.ncbi.nlm.nih.gov/assembly/GCF_000243295.1/
Cape rock hyrax genomic data: https://www.ncbi.nlm.nih.gov/assembly/GCA_004026925.2/
Beluga whale genomic data: https://www.ncbi.nlm.nih.gov/assembly/GCF_002288925.1/
Polar bear genomic data: https://www.ncbi.nlm.nih.gov/assembly/GCF_000687225.1/
Walrus genomic data: https://www.ncbi.nlm.nih.gov/assembly/GCF_000321225.1/
Narwhal genomic data: https://www.ncbi.nlm.nih.gov/assembly/GCA_005125345.1/
(3) Mitochondrial genomes:
Steller's sea cow: https://www.ncbi.nlm.nih.gov/nuccore/NC_040161.1/
Dugong: https://www.ncbi.nlm.nih.gov/nuccore/AJ421723
West Indian manatee: https://www.ncbi.nlm.nih.gov/nuccore/AM904728
African savanna elephant: https://www.ncbi.nlm.nih.gov/nuccore/DQ316069
Asian elephant: https://www.ncbi.nlm.nih.gov/nuccore/DQ316068
Woolly mammoth: https://www.ncbi.nlm.nih.gov/nuccore/DQ316067
Rock hyrax: https://www.ncbi.nlm.nih.gov/nuccore/NC_004919.1

Field-specific reporting

Please select the one below that is the best fit for your research. If you are not sure, read the appropriate sections before making your selection.

[x] Life sciences [ ] Behavioural & social sciences [ ] Ecological, evolutionary & environmental sciences

For a reference copy of the document with all sections, see nature.com/documents/nr-reporting-summary-flat.pdf

Life sciences study design

All studies must disclose on these points even when the disclosure is negative.

Sample size	In this study, we used petrous bone from the Steller's sea cow (Hydrodamalis gigas) skeleton. The sex and age of the animal are unknown. The specimen from Bering Island and is stored in the Museum of the World Ocean (Kaliningrad, Russia). Field number of the sample: Ber/07/Yk-1. Palaeobiological analysis and preservation of the skeleton estimates show that the animal lived several hundred years ago. This sample was selected because of skull petrous bone a good source of endogenous DNA (e.g. PMID: 26086078). The H. gigas individual lived on Bering Island and was a part of the last Steller's sea cow population.
Data exclusions	DNA-libraries Lib1k; Lib3k, Lib4-2, and St11, which had the best fraction of endogenous DNA were used in this study. Other libraries were excused from the deep sequencing plan because low content of endogenous DNA. These libraries were not used for deep sequencing for economic reasons.
Replication	n/a
Randomization	We used only one specimen but with a high content of endogenous DNA. We used several parallel DNA-extractions and selected four libraries with the highest amount of endogenous DNA (>40%) that were used for deep sequencing. Only high-quality SNPs (with p-value < 0.05; coverage > 10X) were used in this study.
Blinding	n/a

Reporting for specific materials, systems and methods

We require information from authors about some types of materials, experimental systems and methods used in many studies. Here, indicate whether each material, system or method listed is relevant to your study. If you are not sure if a list item applies to your research, read the appropriate section before selecting a response.

Materials & experimental systems

n/a	Involved in the study	
[x]	[ ]	Antibodies
[x]	[ ]	Eukaryotic cell lines
[ ]	[x]	Palaeontology and archaeology
[x]	[ ]	Animals and other organisms
[x]	[ ]	Human research participants
[x]	[ ]	Clinical data
[x]	[ ]	Dual use research of concern

Methods

n/a	Involved in the study	
[x]	[ ]	ChIP-seq
[x]	[ ]	Flow cytometry
[x]	[ ]	MRI-based neuroimaging

Palaeontology and Archaeology

Specimen provenance	This study was carried out within the framework of the Federal themes of the Zoological Institute no. AAAA-A19-119032590102-7 “Phylogeny, morphology, and systematics of placental mammals (Alexey N. Tikhonov)
Specimen deposition	The Steller’s sea cow skeleton is housed in the Museum of the World Ocean (Kaliningrad, Russia). Genomic data can be downloaded from National Center for Biotechnology Information (NCBI) database, Bioproject 484555: https://www.ncbi.nlm.nih.gov/bioproject/484555.
Dating methods	The skull bones (including petrous bone) preservation suggested that the animal died in the last years of sea cow population existence on Commander Islands (during the 1760s). In the case of this specimen, it was impossible to conduct radiocarbon dating for this specimen due to the proximity of these dates to the present day.

☐ Tick this box to confirm that the raw and calibrated dates are available in the paper or in Supplementary Information.

Ethics oversight	Ethical approval is not needed because we worked with extinct animal

Note that full information on the approval of the study protocol must also be provided in the manuscript.
