# Supplementary material for: Steller’s sea cow genome suggests this species began going extinct before the arrival of Paleolithic humans
Source: Nat Commun. 2021 Apr 13;12:2215. doi: 10.1038/s41467-021-22567-5 (PMC8044168; doi:10.1038/s41467-021-22567-5)
Supplement: Supplementary file 4 — Description of Additional Supplementary Files [file 41467_2021_22567_MOESM4_ESM.pdf]

Description of Additional Supplementary Files

File Name: Supplementary Data 1
Description: Analysis of single nucleotide polymorphisms (SNPs) in the Steller's sea cow nuclear genome

File Name: Supplementary Data 2
Description: The genes name for the nonsense variants in the Steller's sea cow nuclear genome

File Name: Supplementary Data 3
Description: The genes name for the nonsynonymous variants in the Steller's sea cow nuclear genome

File Name: Supplementary Data 4
Description: The list of the loci that were under positive selection in the Steller's sea cow nuclear genome
